# Treatment of mid-trimester preterm premature rupture of membranes (PPROM) with multi-resistant bacteria-colonized anhydramnion with continuous amnioinfusion and meropenem: a case report and literature review

**DOI:** 10.1007/s00404-021-06319-w

**Published:** 2021-11-18

**Authors:** Michael Tchirikov, Ronja Ocker, Gregor Seliger, Katarina Chaoui, Stefan Moritz, Roland Haase

**Affiliations:** 1 Department of Obstetrics and Fetal Medicine, Clinic of Obstetrics and Prenatal Medicine, Center of Fetal Surgery, University Hospital Halle (Saale), Martin Luther University Halle-Wittenberg, Ernst-Grube Strasse 40, 06120 Halle (Saale), Germany; 2 Department of Neonatology and Pediatric Intensive Care, University Hospital Halle (Saale), Martin Luther University Halle-Wittenberg, Ernst-Grube Strasse 40, 06120 Halle (Saale), Germany; 3 Department of Clinical Infectiology, University Hospital Halle (Saale), Martin Luther University Halle-Wittenberg, Halle (Saale), Germany

**Keywords:** Amnioinfusion, Anhydramnion, 3-MRGN, *E. coli*, Preterm premature rupture of membranes, PPROM

## Abstract

**Purpose:**

Treatment of mid-trimester classic preterm premature rupture of membranes **(**PPROM) with systemic antibiotics has limited success in the prevention of chorioamnionitis, funisitis and fetal inflammatory response syndrome because of very low transplacental passage.

**Methods:**

Here we report a case of PPROM at 18 weeks gestation with anhydramnion colonized by multi-resistant *Escherichia coli* (*E. coli*). A catheter system was implanted at 23/2nd weeks gestation, enabling long-term continuous lavage of the amniotic cavity with Amnion Flush Solution (100 ml/h combined with intraamniotic meropenem application).

**Results:**

The patient gave birth to a preterm male infant at 28/3rd without any signs of infection. In a follow-up examination at 24 months, there was no neurological disturbance or developmental delay.

**Conclusion:**

The classic PPROM with multi-resistant *E. coli* colonization could be treated with continuous amnioinfusion and meropenem.

## Introduction

The ‘classic’ mid-trimester preterm premature rupture of membranes (PPROM) with oligo/anhydramnion is associated with a very high neonatal mortality rate as well as an increased risk of long- and short- term severe neonatal morbidity [1, 2]. The bacteria rapidly colonize surfaces of amniotic membranes, the umbilical cord and the fetus [2]. Expectant management, broad-spectrum antibiotics and antenatal corticosteroids are routinely used in classic PPROM cases with very limited success [1–4]. Gomez et al. [5] reported that antibiotics failed to eliminate the amniotic infection in 83% of PPROM cases.

The lavage effect of continuous amnioinfusion could prevent the fetus and amniotic cavity from bacterial colonization, reduce the inflammatory response mediated by cytokines and protect the neonate from major complications, such as pulmonary hypoplasia, sepsis, cerebral palsy and joint deformities [2, 6–9]. Here we report the management of a classic PPROM and multi-resistant *Escherichia coli* (*E. coli*) (3-MRGN) infection with continuous amnioinfusion and intraamniotic administration of meropenem.

## Case

**Fig. 1:**
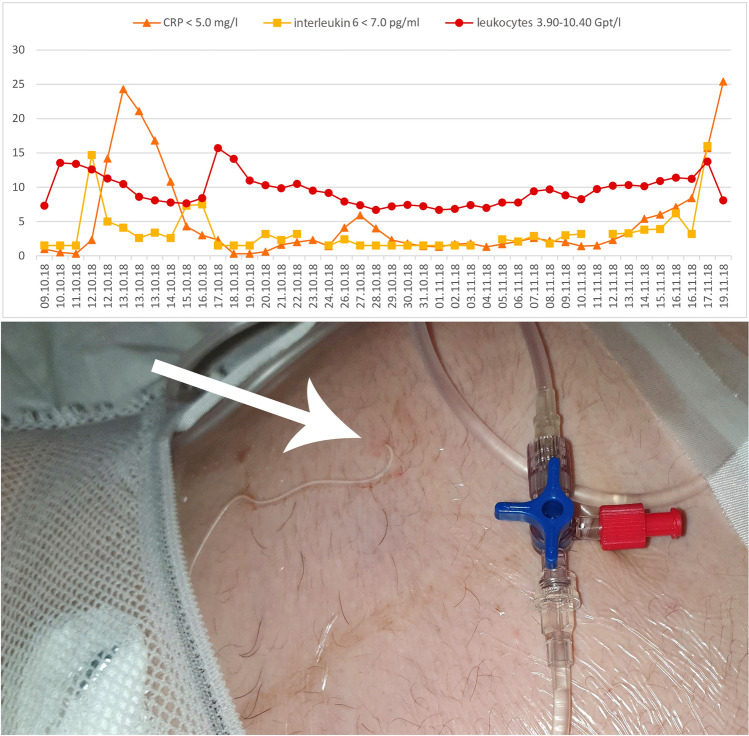
Monitoring of maternal C-reactive protein (CRP), interleukin-6 and leucocytes after preterm premature rupture of membranes (PPROM). The arrow points to the skin’s puncture with the implanted prenatal ‘anker’ catheter

An 18-year-old first gravida was referred to our clinic because of classic PPROM since the 18^th^ week of gestation (WG). On admission at 23/0 WG, she complained about massive loss of amniotic fluid. The PPROM was additionally confirmed by positive test of PAMG-1, (AmniSure®, Qiagen Company, France) and anhydramnion. The estimated fetal weight was 336 g. The patient received a single course of betamethasone (12 mg daily for two days; Celestan®, Essex Pharma, Munich, Germany) for prophylaxis of neonatal respiratory distress syndrome. Ampicillin (1000 mg; intravenous) three times daily (Ratiopharm GmbH, Ulm, Germany) and one application of azithromycin (500 mg; per os) (Hexal AG, Holzkirchen, Germany) were used according to the German guidelines [3]. A cervical bacterial swab was performed weekly (Table 1), C-reactive protein (CRP), leucocytes and Interleukin-6 were monitored and controlled daily (Fig. 1).

**Table 1:** Timeline of antibiotic treatment and diagnostic tests

Days	− 2	− 1	0	1	2	3	4	5	6	7	8	9	10	11	12	13	14	15	16	17	18	19	20	21	22	23	24	25	26	27	28	29	30	31	32	33	34	35	36	*1	*2	*3
Antibiotic treatment																																										
Ampicillin 1 g 3 × 1 iv	x	x	x																																							
Azithromycin po	x																																									
Clindamycin 600 mg 2 × 1 intraamnial			x	x	x	x																																				
Amoxcillin 1 g 3 × 1 po			x	x	x	x	x																					x	x	x	x	x	x	x	x	x	x	x	x			
Metronidazol 500 mg 3 × 1 po				x	x	x	x	x	x	x	x																															
Clarithromycin 250 mg 2 × 1 po				x																																						
Meronem 3 × 1 g iv							x	x	x	x	x	x	x																													
Meronem 500 mg 1xtgl intraamnial								x	x	x	x	x	x	x	x	x	x	x	x	x									x	x	x	x	x	x	x	x	x	x	x			
Ciprobay 400 mg 1xtgl intraamnial																					x	x	x	x	x	x	x	x														
Ciprofloxacin 500 mg 2 × 1 po																			x	x	x	x	x	x	x	x	x	x														
Cefuroxim 1,5 g 3 × 1 iv																																							x	x	x	x
Clont 500 mg 2 × 1 iv																																							x	x	x	x
Microbiological findings of the cervical/vaginal or rectal smears																																										
Extraction	x							x							x	x								x	x			x								x						
Urogenital mycoplasma																																										
*Escherichia coli* (ESBL) 3MRGN	x																											r														
*Enterococcus faecalis* (Gentamicin high-level resistance demonstrated)																x								x	x																	
*Enterococcus faecium* (Gentamicin high-level resistance)	x																																			x						
*Ureaplasma urealyticum*	x																																									
*Candida albicans*																									x											x						
Insertion of catheter			x																																							
New insertion of catheter																	x																									

ESBL, extended spectrum beta-lactamase; i.v., intraveneously; AmpC, AmpC-type β-lactamase; MRGN, multi-resistant gram negative; HBsAg, hepatitis B surface antigen; Anti-HBs, hepatitis B surface antibody; Anti-HBc, hepatitis B core antibody; CMV, cytomegalovirus; Ig, immunoglobulin; r, rectal smear

Appropriate counseling was provided including risks and benefits of preterm birth for the fetus and mother, conservative management versus continuous amniotic infusion via a perinatal catheter system with Amnion Flush Solution (2400 ml daily) combined with intrauterine administration of antibiotics (clindamycin 600 mg, Fresenius Kabi, Bad Homburg, Germany) [2, 6, 7]. The implantation of a perinatal ‘anker’ catheter was performed at the 23/2 WG (Fig. 1, day 0) with the permission of the Martin Luther University Halle-Wittenberg’s ethics committee, and written informed patient consent [2, 7].

At 23/6 WG, a cervical bacterial swab culture showed colonization by multi-resistant 3-MRGN *E. coli* sensitive to meropenem. This antibiotic is listed as Category B in pregnancy [10]. We could not find any information about the intraamniotic use of meropenem and its local effect on the fetus and amniotic membrane. Only a little information was available in relation to meropenem’s effect on neonates [11, 12].However, a low trans-placental passage of meropenem is known [10]. Appropriate counseling was provided with neonatology and infectiology teams including the risks and benefits for fetus and mother. The possibility of intraamniotic meropenem application was discussed with the family before getting written informed consent.

Meropenem (Eberth Pharma, Germany) was administered for ten days at 500 mg/day using the perinatal catheter system into the amniotic cavity and 1000 mg three times/day intravenously (Table 1).

Sensitive to the antibiogram, ciprofloxacin (Ciprobay®, Bayer Vital, Germany) was administered for eight days at 400 mg/day through the into the amniotic cavity and 500 mg twice a day per os. The therapy with meropenem was restarted because of a positive multi-resistance 3-MRGN *E. coli* culture.

The patient gave birth to a male infant via cesarean section at 28/3rd WG (birth weight 1480 g, Apgar score of 7/7/7 at 1/5/10 min, umbilical venous pH 7.49, BE-3) because of increased inflammation parameter Interleukin-6 and CRP without maternal fever. The neonate was transferred to neonatal intensive care unit for further diagnostic and therapeutic management. The newborn required a less invasive surfactant application (Alfeofact®, Lyomark Pharma) followed by non-invasive ventilator support for five days. At admission, Interleukin-6 and CRP values were within normal range. The blood culture as well as throat and umbilical swabs were sterile. The meropenem treatment of the newborn was discontinued at the third day because no clinical signs of neonatal infection were displayed during the period of hospitalization. The neonate was discharged weighing of 3606 g in good condition. The histological examination of amniotic membranes and placenta (weight 405 g, size 14 cm × 13 cm × 3 cm), showed no signs of chorioamnionitis. Follow-up examination at 24 months confirmed normal development without any signs of neurological disturbance.

## Discussion and literature review

The very early delivery (prior to 28 weeks of gestation) of premature babies is a worldwide health problem also in developed countries as it is associated with a very high neonatal mortality rate as well as an increased risk of long- and short-term severe neonatal morbidity, physical and developmental disabilities, including chronic respiratory disease (RDS), neurodevelopmental or behavioral effects (impairment of visual/hearing/executive functioning, global developmental delay and psychiatric/behavioral sequela) and cardiovascular diseases [2, 13, 14].

Mid-trimester preterm premature rupture of membranes (PPROM) affects women during the second trimester of their pregnancy. About 0.4–0.7% of all pregnancies are affected by this complication [2]. The causes of the mid-trimester PPROM are multifactorial including local infiltration by bacteria with reaction of pro-inflammatory cytokines, pathologic anatomical remodeling of the amniotic membrane (contribution of MMPs), invasive procedures and fetoscopic surgery, genetic and iatrogenic factors, smoking, vaginal bleeding etc. [2].

Altered membrane morphology including marked swelling and disruption of the collagen network which is seen with PPROM can be triggered by bacterial products or/and pro-inflammatory cytokines [2]. The “classic PPROM” with oligo/ anhydramnion is associated with short latency period and worse neonatal outcome compared to similar gestational aged neonates delivered without antecedent PPROM [2].

Even though the survival rate of premature infants born before 28 weeks of gestation has improved significantly over the last several decades, extreme preterm delivery is still often associated with subsequent neonatal death prior to 1 month of age [15, 16]. Crane et al. [13] published the outcome of infants in Canada delivered at 23 weeks' gestation. The perinatal mortality was 89.9% and of live born neonates admitted to NICU neonatal death occurred in 63.8%. Among survivors at discharge, the rate of severe brain injury was 44.0%, of retinopathy of prematurity 58.3%, and of any serious neonatal morbidity 100% [13]. More than 40% of surviving neonates following PPROM prior to 25 weeks of gestation develop bronchopulmonary dysplasia (BPD) [2].

Prolonged anhydramnion after PPROM is associated with a four-fold increased risk of composite adverse outcomes, including death, BPD, severe neurological disorders, severe retinopathy, when compared to an age-adjusted control group [15, 16].The reaccumulation of the amniotic fluid could improve the neonatal outcome [17].

Spreading of bacterial products and/or pro-inflammatory cytokines trigger alteration of membrane morphology including marked swelling and disruption of the collagen network which is seen with PPROM [2].

Along with expectant management and antenatal corticosteroids, broad-spectrum antibiotics are routinely used with relative limited success in mid-trimester PPROM to prevent bacteremia, chorioamnionitis and FIRS [2, 3]. The trans-placental transfer of antibiotics is depend to used medicine. Some antibiotics with large molecules pass through the placental barrier worse compared to penicillin-group [18–21]. The amniotic membranes and the umbilical cord do not have an effective capillary net and the antibiotics from maternal circulation do not directly reach the bacteria-colonized surfaces in sufficient concentrations [2].

In our case, the pregnancy was complicated by multi-drug resistant *E. coli* colonization after the classic PPROM with anhydramnion. *E. coli* is a bacteria, often isolated to a hospital setting [22]. We report the first combination of continuous amnioinfusion with Amnion Flush Solution 100 ml/h and intraamniotic meropenem application for the treatment of PPROM with multi-resistant *E. coli* colonization and prophylaxis of FIRS. The i.v. administration of meropenem to the patient was discussed. The majority of investigators as well the patient took a decision to perform a combined i.v. and intraamniotic meropenem applications. In our opinion, the systemic administration of the antibiotic as well as the change of antibiotic related to the antibiogram is a general problem of the sufficient PPROM treatment and should be discussed with specialists in any specific case.

A literature search in Pubmed for “amnioinfusion” in different search contexts revealed a number of 82 hits (including some doubles) resulting in 28 relevant publications, consisting of reviews, randomized clinical trials (RCTs), non-randomized/observational clinical trials and case studies, outlining the state of the art of serial or continuous transabdominal amnioinfusion in most cases using standard infusion solutions who are used off-label in this regard.

Roberts et al., demonstrated that repetitive transabdominal amnioinfusions with Ringer’s lactate solution did not improve the perinatal outcome of PPROM patients. In their study, 14 neonates died in the amnioinfusion group versus nine in the control group [23]. Van Kempen et al. [24] (PPROMEXIL-III trial) also did not find any reduction in perinatal mortality after repetitive amnioinfusion with Ringers lactate solution in women with mid-trimester PPROM with oligohydramnion.

A group from Japan demonstrated 2020 that continuous amnioinfusion with Ringer’s lactate solution (40 ml/h) significantly increased the PPROM delivery interval by 2 weeks without improving the neonatal outcome [8]. This could be partly explained by a very low ‘flush-out’ effect because of the high frequency of catheter dislocation (60%) and lack of pump infusion in many cases [8, 9].

Fetal skin in second trimester is still very thin and permeable. It is a matter of common knowledge that the fetus swallows and inspirates/expirates amniotic fluid.

Gilbert and Brace published that the fetus swallows 200–250 ml/kg/day amniotic fluid [25].Continuous amnioinfusion with Normal saline solution significantly increased plasma Na^+^ and Cl^−^ concentrations in fetal sheep [26].

The amniotic fluid is a very complex hypoosmotic solution with an alkaline pH, low concentration of the elements Cl^−^, K^+^ and Na^+^, presence of trace elements, growth factors and surfactants etc. [27]. In our opinion, the change of physiological fetal surroundings for a long period using amnioinfusion with simple electrolyte solutions could destroy in all probabilities the fetal programming. Osmotic demyelization disorders, central pontine and extra-pontine myelinolysis are well known [28]. Chhabra et al. [29] described the extra-pontine myelinolysis induced by hypernatremia. The combination of very immature brain-blood barrier of the fetus with the permeable skin and swallowing of relative large amount of electrolyte solution deteriorates the fluctuations of the NaCl concentration of the fetal brain. This could explain the high neonatal mortality rate after serial amnioinfusion in the Roberts et al., study and very moderate success of continuous amnioinfusion using Ringer’s lactate solution (inappropriate to human amniotic fluid) described in the retrospective Japanese studies [8, 9, 23, 30].

Tchirikov et al. [31] described the use of pump-regulated (100 ml/h) continuous intraamniotic lavage using simple electrolyte solutions through the catheter without fixation since 2008. The dislocation rate of the catheter was very high, until the catheter was improved by an anchor fixation [7]. In our case we decided to replace the anchor catheter after two weeks avoiding the possible local skin inflammation. Last 2 years we replace the catheter normally after one month. In cases of the local skin reaction it must be replaced, too. The patients in whom Jonosteril® (Fresenius Kabi GmbH, Germany), Sterofundin® and isotonic NaCl (B. Braun AG, Melsungen, Germany) or lactated Ringer’s solution (Baxter, Germany) were used, reported significantly increased diuresis, probably trans-planetary, triggered by a fetal response to increased NaCl, fluctuation of osmolality and missing microelements also referred to as ‘Salzgurken’ effect [2]. The electrolyte solutions for the continuous amnioinfusion were replaced 2012 by artificial amniotic fluid (now Amnion Flush Solution, CE0483, Serumwerk AG, Bernburg, Germany) without any adverse events [2, 6, 7]. In 2020, the German Federal Ministry of Education and Research funded a prospective multicentre randomized study investigating the effect of continuous amnioinfusion (22/0-26/0 WG) with Amnion Flush Solution compared with the standard PPROM therapy (ClinicalTrials.gov: NTC04696003).
